# Longitudinal Vibrations in the Organ of Corti are Limited to Its Core

**DOI:** 10.1007/s10162-025-01017-9

**Published:** 2025-11-18

**Authors:** Sebastiaan W. F. Meenderink, Marcel van der Heijden, Wei Dong

**Affiliations:** 1 VA Loma Linda Healthcare System, Loma Linda, CA 92374 USA; 2 Department of Neuroscience, Erasmus MC, 3000 CA Rotterdam, the Netherlands; 3 Department of Otolaryngology—Head and Neck Surgery, Loma Linda University Health, Loma Linda, CA 92350 USA

**Keywords:** Cochlear mechanics, Gerbil, Longitudinal motion, Optical coherence tomography, Vibrometry

## Abstract

**Purpose:**

The goal of this research was to determine where in the organ of Corti (ooC) sound–induced, longitudinal vibrations occur, and how they depend on the health of the cochlea.

**Methods:**

Sound-evoked vibrations of the ooC in the cochlea’s middle turn of adult anesthetized gerbils were measured using optical coherence tomography (OCT). Vibratory responses, evoked with acoustic tone complexes, were recorded at multiple, closely spaced (20 μm), tonotopic locations which changed the “viewing angle” of the vertical OCT beam re*.* the longitudinal motion. After spatial alignment of the responses, within-ooC regions exhibiting sound-induced longitudinal motion were identified from a conspicuous 180° phase flip.

**Results:**

Longitudinal motion was restricted to the outer hair cells (OHC), Deiters’ cells and the tunnel of Corti (i.e., the ooC’s “core”). They were frequency and level-independent but did depend on the ear’s metabolic state; after death, they disappeared.

**Conclusion:**

There can be little doubt about the presence of longitudinal motions within the cochlea. Their disappearance postmortem and spatially restricted occurrence suggest these longitudinal vibrations arise from active processes within the OHC. Whether this involves cycle-by-cycle feedback or some other, as-of-yet undetermined, mechanism remains to be resolved.

**Supplementary Information:**

The online version contains supplementary material available at 10.1007/s10162-025-01017-9.

## Introduction

The cochlea is a spiraled, fluid-filled tube that holds the sensory epithelium in which specialized (hair) cells transduce sound energy to electrical signals [1]. Prior to transduction, the sound energy—which enters the cochlea near its base—is distributed along the sensory epithelium such that high (low) frequencies maximally excite hair cells located near the cochlea’s base (apex). This distribution involves a relatively slow mechanical, traveling wave during which substantial conditioning (e.g., compression) of the signal occurs. This pre-transduction conditioning is essential to achieve the exquisite sensitivity, selectivity and dynamic range of the mammalian ear, but its underlying mechanisms are far from fully understood.

Optical coherence tomography (OCT) has become the most popular technique to directly measure these cochlear mechanics [2]. It can both visualize the different structures within the inner ear and measure the minute (< 1 nm) motions inside the organ of Corti (ooC) that are associated with hearing. Its application to hearing research immediately led to a myriad of new, and unanticipated, data showing that intra-ooC motions are complex and do not simply follow the transverse, “up-and-down” displacement of the basilar membrane (BM). For example, outer hair cell (OHC) responses were less tuned and exhibited nonlinear responses over a wider frequency range than the BM (e.g., [3–5]). Moreover, the intra-ooC motions were 3-D, in which transverse vibrations are combined with, or even dominated by, significant motions in the radial and/or longitudinal direction [6–9].

As with all interferometric techniques, OCT only measures that part of the motion that is parallel to its optical axis, and the amplitude, phase and direction of the motion can only be determined by combining recordings from three or more differently angled measurement beams. Although doable [9, 10], this remains difficult when applied to cochlear mechanics. The cochlea’s location within the skull makes it nigh impossible to create sufficiently large angle differences between the beams. A large angular range is needed because the “true” vibration direction and magnitude is reconstructed from changes in the measured vibration amplitudes obtained with different beam angles. In the absence of noise, any angle variation suffices, but at smaller signal-to-noise ratios, it is unrealistic to reliably measure the small amplitude changes associated with small beam-angle variations. In addition, it is also difficult to accurately register a single recording location across the measurement angles without an optical model of the cochlea, although the use of intracochlear, physiological responses for registration can (partially) alleviate the latter problem [9].

As an alternative to reconstructing the 3-D motion from multiple measurement angles at one location, we recently used the natural curvature of the cochlea to vary the angle between the OCT measurement beam and the longitudinal axis of the BM to look for a sign of motion along the longitudinal (tonotopic) axis of the cochlea [8]. Such recordings are not suitable for 3-D reconstruction of the motion (as measurement angle co-varies with location), but we did find that the phase of the OHC responses flipped 180° when this “measurement angle” changed sign (see also [7]). This is as expected in the presence of significant longitudinal motion. Moreover, we found that the OHC response was 90° out-of-phase with the BM, indicating that the longitudinal motion was elliptical in nature. We interpreted this to signify that the sound induced motion within the ooC arose from a traveling surface wave that exhibited a strong longitudinal motion component. In terms of fluid dynamics, this suggests a “long wave regime”, familiar from waves on shallow water.

Based on the ooC’s anatomy, we hypothesize that longitudinal motion primarily occurs near the OHC-Deiters’ cell junction and within the tunnel of Corti. The less dense packing of cells in those regions provides the “degree-of-freedom” to move in the longitudinal direction. In this study, we tested this hypothesis in the second turn of the gerbil cochlea. In each cochlea, we recorded multiple high-resolution phase vibrometry maps that were spaced along the longitudinal axis. To assess the importance of physiological condition for the occurrence of the longitudinal motion, we varied stimulus intensity and compared the responses of healthy, tone-damaged, and postmortem cochleae.

## Methods

All methods were like those used in [8, 11]. Briefly, recordings were from adult Mongolian gerbils (*M. unguiculatus*). To illustrate the findings, data from four animals, all female, are included in the manuscript. Identical results were obtained in more animals (of either sex) as the described recordings are routinely made in our laboratory. Animals were deeply anesthetized (ketamine/xylazine i.p. at 80/10 mg/kg, respectively), and their body temperature was kept at ~ 38 °C using a heating pad (Harvard Apparatus). A ventrolateral approach surgically exposed the left cochlea, which itself was not compromised. The ipsilateral pinna and the cartilaginous ear canal were resected, and the tip of a microphone and transducer assembly (ER-10X, Etymotic Research) was sealed to the exposed ear canal using grease for closed-field configuration. Animals were not allowed to recover from anesthesia and were euthanized by anesthetic overdose.

Images and vibratory responses were recorded from the ooC in the middle turn of the cochlea using a spectral domain OCT system (Thorlabs Telesto III TEL321C1 equipped with an LSM04 objective). Optical spectra were acquired with a sample frequency of 24.4 kHz. From each of the spectra, we (1) subtracted the spectrum of the light source, (2) corrected for the non-uniform wavenumber intervals in the spectrometer using piecewise cubic spline interpolation, and (3) multiplied with a window before converting them into depth-resolved, axial information (A-line) using Fourier analysis. OCT images (B-scans) were constructed by concatenating the brightness profiles from multiple, adjacent A-lines that were obtained while scanning the OCT beam. Here, the scan path was traversed multiple (> 10) times, and the brightness profiles for each A-line were averaged to improve the quality of these images.

For vibrations measurements (M-scans), the phase information of the A-lines was used. With the OCT beam at a fixed position, A-lines were recorded while an acoustic stimulus was presented (RX6: Tucker Davies Technologies system III) to the ear. These stimuli were 12-s tone complexes that were “zwuis” for both 2nd and 3rd-order distortions [12] and consisted of 27 frequency components (0.4–4.4 kHz), each with a random starting phase. With this number of frequency components, the total power of the stimulus was 14 dB larger than that of its constituents. In two animals (Figs. 6 and 7) the cochlea was damaged by exposure to 106 dB SPL tone(-s). In one animal we used 1, 2, and 8-kHz tones, presented sequentially, while the other was exposed to a single 1-kHz tone. The exposure was titrated in ~ 30-s increments until the 2*f*_1_–*f*_2_ DPOAEs (*f*_2_/*f*_1_ = 1.25, *L*_1_ = *L*_2_ = 50 dB SPL) had largely disappeared into the noise floor for *f*_2_ < 5 kHz.

At each depth, vibratory responses at the stimulus frequencies were extracted from the recorded signals using Fourier analysis. No artifact rejection was employed. A frequency component was considered above noise when a Rayleigh’s test for uniformity indicated significant (*p* ≤ 0.001) phase locking to the stimulus [13]. Responses not meeting this criterium were excluded from further analysis and are not shown throughout the manuscript.

The OCT system was controlled (e.g., mirror position, data acquisition) by custom software in C^#^ (Visual Studio, Dev14), while stimulus generation, hardware synchronization, and signal analysis were implemented in Matlab (MathWorks, R2018b).

### Ethics

The care and use of animals was in accordance with guidelines of, and approved by, the Institutional Animal Care and Use Committee (IACUC) of the VA Loma Linda Healthcare System (Animal Welfare Assurance Number A3723-01; protocol 1,727,721–8).

## Results

**Fig. 1:**
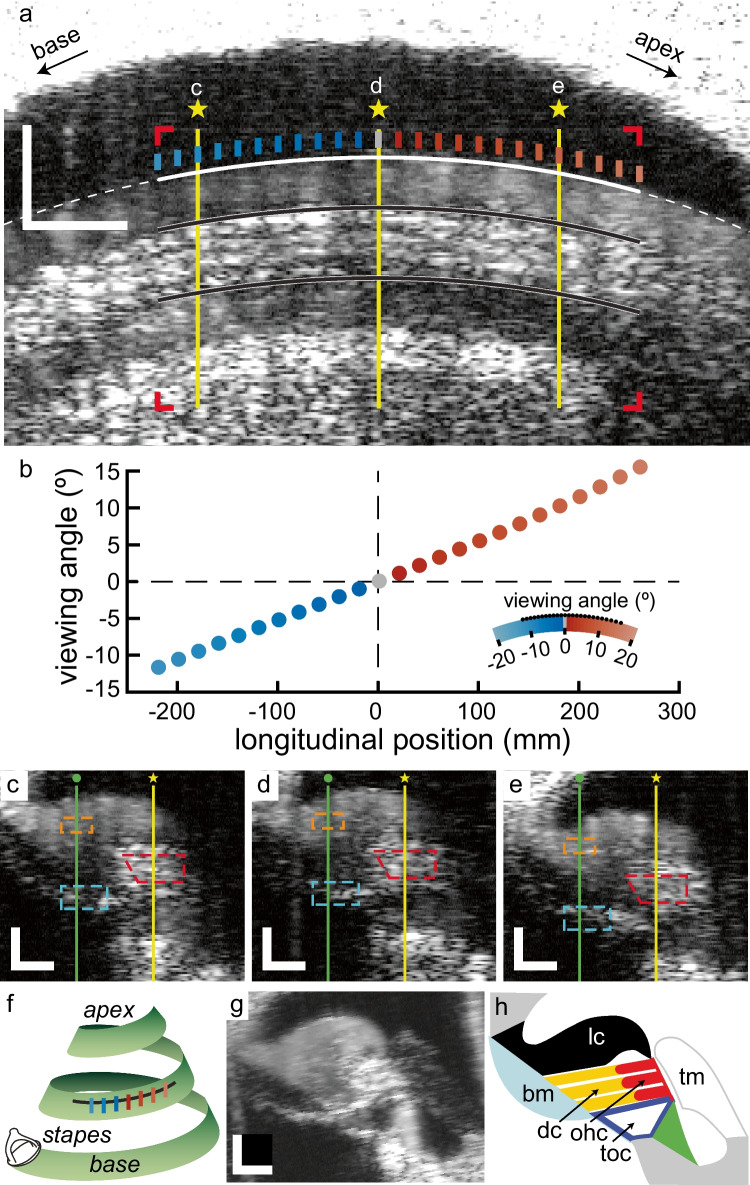
Overview of recording positions and anatomical identification. **a** B-scan along the tonotopic axis in the second turn of the gerbil cochlea that transects the organ of Corti at the level of the OHC. At each of the 25 longitudinal locations shown (vertical line segments, 20 μm apart), radial B-scans and radial phase vibrometry maps were made. The cartoon in panel (**f**) shows the orientation of the longitudinal B-scan (black line) and these radial maps (colored lines) within the second turn of the cochlea. An ellipse (white line) was fitted to the boundary between the tectal cells and scala media and used to define the “viewing angle” of the recordings (see text). Red corner markers define the vibrometry region-of-interest; solid black lines are translated repeats of the fitted ellipse. The yellow lines and * mark the positions of the radial scans in panel c–e. Scalebars: 100 µm. **b** Viewing angles of the 25 positions marked in panel a, plotted against the longitudinal distance along the ellipse (with the origin at the zero-angle position). The abscissas of panels (**a**) and (**b**) are aligned. **c–e** B-scans showing the radial sections at the positions marked in panel (**a**). In each panel, the yellow line and * mark the radial position of the B-scan in panel (**a**). The green line and • indicate a radial position intersecting with the BM. Longitudinal vibrometry data for these two radial positions are shown in Fig. 3. Three regions are delineated: the BM (cyan contour), the OHC region (red contour) and part of the LC (orange contour). Scalebars: 50 µm. **f** Cartoon of the spiraling cochlea (green) and the orientation of the longitudinal B-scan (black line) and radial B-scans/maps (colored lines) within its second turn. **g** Radially oriented B-scan obtained after removing the overlying bony wall of the cochlea. This image is at a similar tonotopic location as shown in panel (**c**), but from a different cochlea that was not used for vibrometry. **h** Annotated diagram of the anatomical structures in panel (**g**)

The vibrometry data of this study consists of volumetric M-scans (i.e., combination of multiple M-scans, each one obtained at a separate A-line) obtained from the second turn of the gerbil cochlea. The volume scans can either be presented in longitudinally or radial sections, and we will use both views to analyze salient features of the vibrations. Figure 1 illustrates the organization of the scans and the anatomical structures of interest.

Figure 1a shows an OCT-based brightness image (B-scan). The cochlea’s orientation was such that this B-scan was parallel with the tonotopic axis (Fig. 1f) and transected the ooC at the level of the three rows of outer hair cells (OHC; marked by the two curved black line segments in part of the image). Above the OHC region, the imaging plane contained cross sections of the lateral compartment or LC (i.e., tectal cells and Hensen cells), scala media and the bony wall of the cochlea. Below the OHC, the tunnel of Corti and osseous spiral lamina were visualized. An ellipse was fitted to the boundary between the LC and scala media (white line) to serve as a proxy for the BM and its curvature in the direction of the tonotopic axis. It was used to define the OCT's “viewing angle”, calculated as the angle between the ellipse’s normal line and the vertical OCT beam [8]. Figure 1b shows the resulting viewing angles for the 25 longitudinal positions, spaced 20 μm apart, indicated by the colored, vertical lines in Fig. 1a. For each of these positions, a radially oriented B-scan transecting the ooC was made, enabling the identification of the anatomical structures. Three of such B-scans are shown in Fig. 1c–e. Although BM orientation in the longitudinal direction systematically varied with longitudinal position (Fig. 1b), it did not in the radial direction. In the radial B-scans obtained at the different tonotopic locations, we measured the BM angle (re. vertical) to be 32–38° for the different longitudinal positions. Although clearly distinguishable in these B-scans, the anatomical structures within the ooC are more easily identified in a B-scan obtained after removing the overlying bony wall of the cochlea (Fig. 1g; a schematic diagram of the anatomical structures is shown in Fig. 1h). The three dashed frames in Fig. 1c–e delineate OHC (red), BM (cyan), and LC (orange) regions. This delineation was done in the B-scans for each of the 25 longitudinal positions, allowing the accurate analysis of OHC, BM, and LC phase as a function of longitudinal position. These three regions of interest (ROI) were defined so that they include pixels with similar phase responses (see also [11] and Fig. 4). Also indicated in Fig. 1c–e are two radial beam positions (vertical lines). The yellow line transects the OHCs and corresponds to the B-scan of Fig. 1a, while the green line transects the BM. No longitudinally oriented B-scan was recorded at this radial position. These two radial positions were used for the “longitudinal phase vibrometry maps” shown in Fig. 3.

**Fig. 2:**
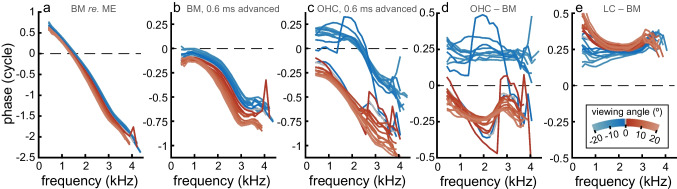
Phase response curves for selected regions within the organ of Corti. **a** Basilar membrane phase (relative to middle-ear response at the umbo) for the 25 recording locations marked in Fig. 1a. **b** An additional 0.6 ms was subtracted to better reveal the tonotopic dependence and frequency dependent group delays. Differences between curves come from the tonotopic organization. The divergence between the curves above 2 kHz reflects the slowing down of the traveling wave near best frequency. **c** Phase for the OHC region, also with 0.6 ms subtracted re. the middle-ear response. Phase difference between the BM and **d** the OHC region and **e** LC. In all panels, phase data were taken from the regions-of-interest as marked in Fig. 1c–e. Using these averaged responses versus single-pixel data yielded similar results (see also Online Resource Fig. 3). The curves are color-coded by viewing angle (key in panel e). The BM phases as shown here are used as reference data for subsequent figures. Stimulus: multitone with 50 dB SPL/frequency component

Figure 2 shows phase-vs.-frequency curves of these 25 longitudinal positions (see Fig. 1a). Each single curve represents the response, averaged over all pixels within each ROI outlined in Fig. 1c–e, to a multitone stimulus (see Methods). When normalized to the middle ear, the phase-vs.-frequency curves of the BM (Fig. 2a) were dominated by a steep downward trend that reflects a common group delay of ~ 800 μs. To bring out the differences between the curves, we show the same data (Fig. 2b), but now compensated for a 600-μs delay [14]. The systematic increase of phase lag with longitudinal position reflects the traveling wave delay which accumulates from base to apex. The divergence of the curves above ~ 2 kHz reflects the slowing down of the wave when it approaches the best frequency.

Figure 2c shows the phase of the OHC vibrations, also compensated for a 600-μs delay. This set of curves reflects an abrupt transition; an ~ 0.5-cycle jump that separates the more basal positions (negative viewing angles) from the apical ones (positive viewing angles). The jump occurred at a viewing angle between − 6 and 0°. Within each of the two groups of curves, the same traveling wave trends are visible as in the BM. The phase jump itself is quantified by plotting the phase difference between the OHC and BM (Fig. 2d). This subtraction cancels the common phase trend stemming from the wave propagation. Basal to the jump, the OHC motion led BM motion by ~ 0.25 cycle; apical to the jump a 0.25-cycle lag was observed. This dependence of OHC re. BM phase on viewing angle was interpreted to signify the existence of longitudinal motion within the OHC region that dominates the recorded response [7, 8]. In this interpretation, the phase flip occurs at the position for which the viewing angle is perpendicular to the spatial direction of the vibrations. In the LC, such phase dependence on viewing angle was not seen; there the phase (re. BM) was largely constant around 0.25 cycle with viewing angle (Fig. 2e).

**Fig. 3:**
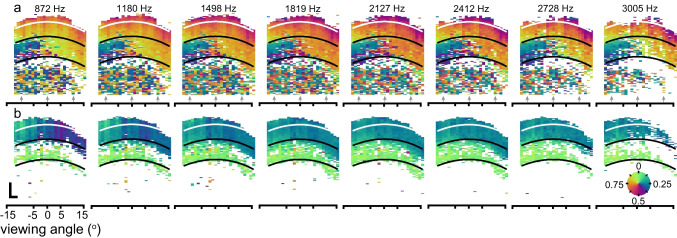
Longitudinal phase vibrometry maps at different frequencies. **a** Maps at different stimulus frequencies for the longitudinal section shown in Fig. 1a and marked by the yellow line in Fig. 1c. **b** Similar, now for the longitudinal section that sections the organ of Corti at the level of the basilar membrane (green line in Fig. 1c). In each map, phase is normalized to the BM phase at the corresponding longitudinal position (see Fig. 2). The white and black lines are the same as the fitted ellipse and its repeats in Fig. 1a. Scalebars (50 µm) and legend for vibrometry phase (in cycle) apply to all maps. For phase, counterclockwise rotation indicates lag. Stimulus: multitone with 50 dB SPL/frequency component

We analyzed the spatial features of the phase flip using phase vibrometry maps. Figure 3a shows a series of such maps based on the longitudinal cross section at the level of the OHCs (cf. B-scan in Fig. 1a; yellow line in Fig. 1c). The phase in these maps was normalized to the (place- and frequency specific) phase of the BM response (Fig. 2a) to remove effects of the traveling wave. With this normalization, maps for all frequencies showed a similar phase pattern in the OHC region (marked by curved black line segments) that changed with viewing angle. For comparison, longitudinal phase vibrometry maps at the level of the BM and LC (cf*.* green line in Fig. 1C) are shown in Fig. 3b, again normalized to BM phase. The phase in this cross section lacks the phase flip; in fact, phase varied little with viewing angle and frequency.

**Fig. 4:**
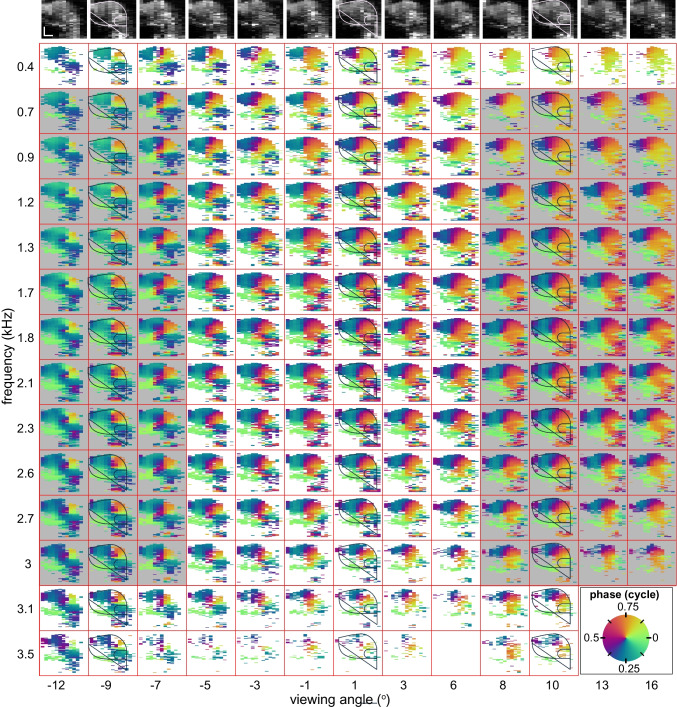
Collage of radial phase vibrometry maps at different frequencies and viewing angles. Series of B-scans (top row), each one obtained at a different viewing angle. These B-scans were reconstructed from the (depth-resolved) mean intensities of the reflected light during the M-scans that yielded the phase vibrometry maps. Each of these phase maps was obtained for a different stimulus frequency (ordinate) and viewing angle (abscissa). In each map, the phase is normalized to the frequency and location-specific BM phase. Gray shaded areas contain maps that were averaged in Fig. 5c, d. Counterclockwise phase rotation (see legend) indicates lag. Scalebars (50 µm) in upper-left corner apply to all. Stimulus: multitone with 50 dB SPL/frequency component. In three of the columns, a coarse structural outline of the organ of Corti is superimposed on the data for visual guidance. See Fig. 1g, h for more details on the organ’s anatomy. The corresponding amplitude data are shown in Online Resource Fig. 1

To delineate the region within the ooC that featured the 0.5-cycle phase flip with viewing angle (and where recordings thus primarily quantify longitudinal motion), we recorded radially oriented phase vibrometry maps at each of the 25 tonotopic locations. Figure 4 shows these maps, again normalized to the place- and frequency-specific BM phase, for a representative subset of frequencies and locations (See Online Resource Fig. 1 for corresponding amplitude data). With the BM normalization, most frequency dependence in the maps from each tonotopic location was removed. That is, all maps within each column of Fig. 4 exhibited a similar phase pattern, even though the stimulus frequencies spanned more than three octaves around BF = 1.8–2.3 kHz. The frequency independence of these (relative to BM) phase patterns alone does not indicate that the relative ooC motion is also independent of frequency; this would only be the case if both the relative phase and amplitude of the vibrations were frequency invariant. It is now well established [2] that the BM and OHC tuning curves exhibit a different frequency response both in the high-frequency base [2–5, 7] and this region [11] of the cochlea.

**Fig. 5:**
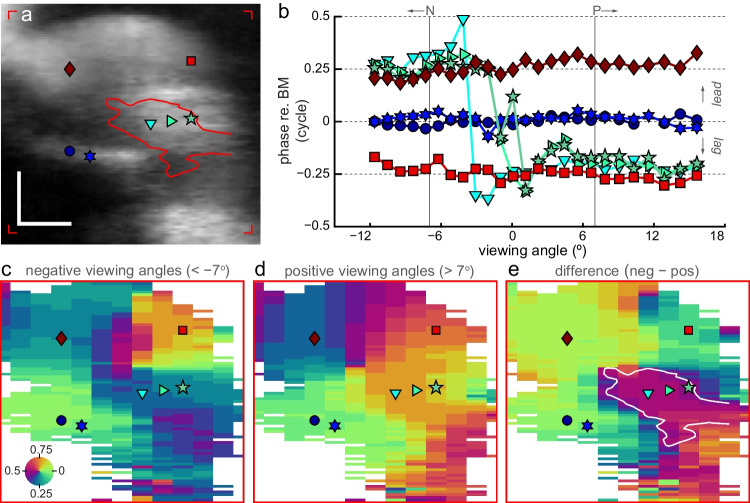
Localizing longitudinal motion within the organ of Corti. **a** B-scan, averaged over 25 tonotopic locations after alignment. Symbols give pixels for which phase-vs.-tonotopic location are shown in (b), red corner markers indicate vibrometry region-of-interest. Scalebars: 50 µm. See Fig. 1h for an annotated diagram of the anatomical structures. **b** Phase-vs.-viewing angle for marked pixels in (**a**). Phase is re. BM phase and averaged for stimulus frequencies between 0.7 and 3 kHz. Vertical lines (marked *N* and *P*) give viewing-angle boundaries used to calculate mean phase vibrometry maps in (**d**, **e**). Mean phase vibrometry maps for **c** negative (< − 7°) and **d** positive (> 7°) viewing angles, averaged across tonotopic locations and stimulus frequency (0.7 ≤ *f*_stim_ ≤ 3 kHz). **e** Difference between the negative and positive phase vibrometry map. White line is a contour at 0.4 cycle, it is shown as a red line in (**a**) where it outlines the Deiters’ cells and the basal part of the outer hair cells. Red boxes around (**c**–**e**) give vibrometry region-of-interest. Stimulus: multitone with 50 dB SPL/frequency component

To obtain a more precise anatomical frame-of-reference for these radial phase vibrometry maps, we used their corresponding B-scans (Fig. 1c–e, see also Fig. 4, top row). These showed that within the volumetric M-scan (covering 0.48 mm along the tonotopic axis), the shape, orientation and dimensions of the ooC varied little with tonotopic location, not unlike the swirl in a properly rolled Bûche de Noël or Yule Log Cake. We used this anatomical invariance along the tonotopic axis to align the B-scans using translation, resulting in the averaged cross-sectional B-scan shown in Fig. 5a. The same translations were then applied to the phase maps. Effectively, this “straightens out” the volumetric M-scan, so that the effect of viewing angle on phase can be evaluated for each position/pixel within the ooC. This is shown in Fig. 5b for seven positions within the ooC: on the BM (blue symbols), within the OHC region (greenish symbols), and within the LC (red symbols), respectively. Since phase was largely independent of stimulus frequency after normalizing to the average BM response (Figs. 3 and 4), we used the frequency averaged (0.7 ≤ *f*_stim_ ≤ 3 kHz) response at each viewing angle in this plot. As expected, the (normalized) phase for the two positions on the BM was close to zero and did not vary with viewing angle. Similarly, the two positions within the LC had constant phase across viewing angle, although they moved 0.25 cycle out of phase re. the BM. Phase for the three positions within the OHC/Deiters’ cell region was not constant with viewing angle but showed the 0.5-cycle phase-flip associated with longitudinal motion. As was observed in Figs. 2 and 4, OHC motion led the BM response by ~ 0.25 cycle for negative viewing angles but lagged by 0.25 cycle when viewing angle was positive.

At this point, the observations on the phase patterns may be summarized as follows:

- Once normalized to BM phase, the phase vibrometry maps at each viewing angle show little variation across frequency (columns in Fig. 4)
- When present, the effect of viewing angle on response phase appears to be bimodal, with a transition near 0° (Fig. 5b)

The uniform character of the data justifies an additional averaging procedure, namely over frequency (700–3000 Hz) and two ranges of viewing angle (marked by *N* and *P* in Fig. 5b), namely angles < − 7° and angles > 7°, respectively. Specifically, each of the two sets of phase vibrometry maps indicated in Fig. 4 by the gray areas were averaged. These average maps are shown in Fig. 5c (negative viewing angles) and Fig. 5d (positive viewing angles). The location of the phase flip within the ooC was analyzed by taking the difference of these two average maps, the resulting phase difference map is shown in Fig. 5e. It shows that the ooC region in which the 0.5-cycle phase flip occurred was limited to the Deiters’ cells, (the basal aspects of) the OHC and the tunnel of Corti, as indicated by the white contour. This shows that longitudinal vibrations in the ooC are limited to its “core”.

**Fig. 6:**
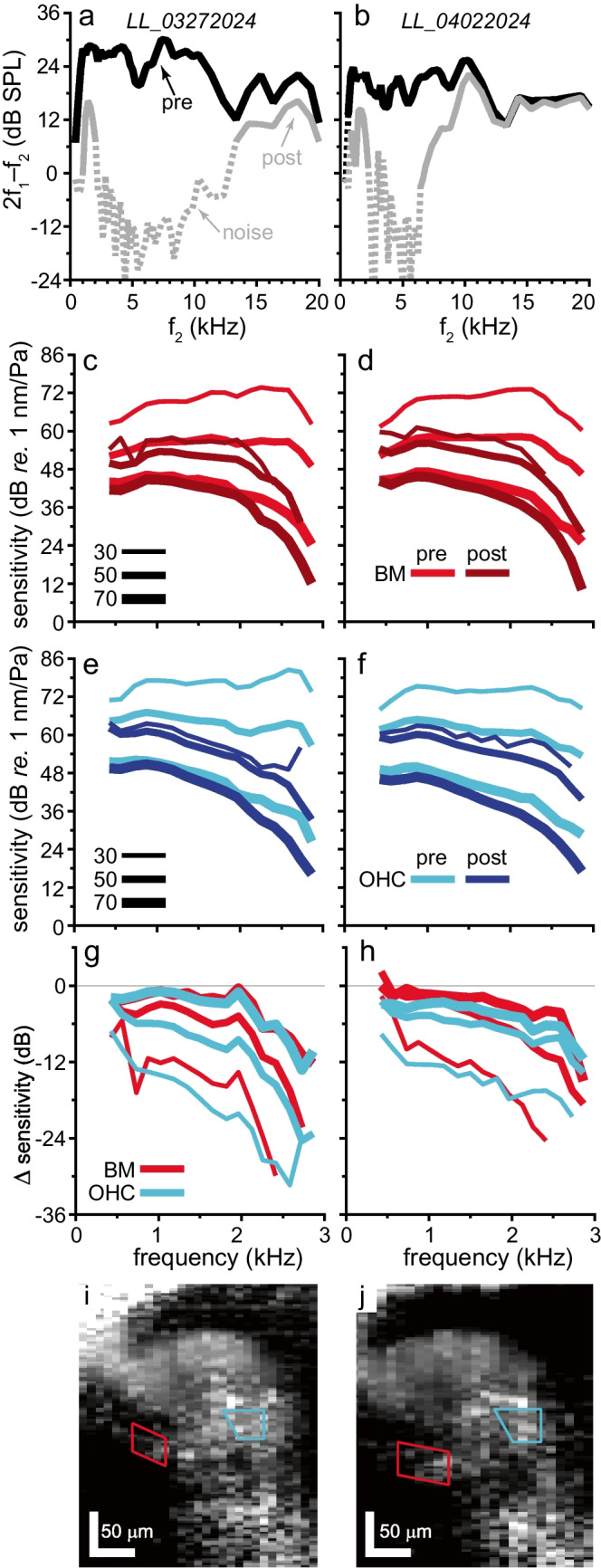
Effect of overexposure on DPOAEs and BM gain curves. **a, b** DPOAE amplitudes (*L*_1_ = *L*_2_ = 55 dB SPL, *f*_2_/*f*_−1_ = 1.25) recorded from two animals (columns) before (black line) and after (gray lines) damaging their cochlea by exposure to 106 dB SPL tone(-s) at (**a**) 1, 2, and 8-kHz tones, presented sequentially, or (**b**) a single 1-kHz tone. Dashed lines indicate that DPOAEs could not be resolved from the noise floor (+ 6 dB SNR). Postmortem, no DPOAEs that exceeded the noise floor were observed (not shown). Gain curves from **c**, **d** the BM and **e**, **f** the OHC region before and after tone exposure. **g**, **h** Change in sensitivity, calculated from the data in (**c**–**f**), is level dependent, but not obviously different between the BM and OHC region. **i**, **j** B-scans, obtained during vibrometry, with regions-of-interest for the BM (red) and the OHC region (cyan) superimposed. Mean responses for these regions are shown in panels (**c**–**h**). All data are for the animals shown in Fig. 7 middle row and bottom row, respectively

The bimodal dependence of the phase patterns with viewing angle indicates that a comparison of responses from two longitudinal locations only is sufficient to observe the phase flip, provided that these locations are at sufficiently negative and positive viewing angles (see also Online Resource Fig. 2). We exploited this simplified, more efficient comparison to assess the effects of inducing cochlear damage using intense tones. Before considering the phase vibrometry maps, we illustrate the primary effects of overstimulation in Fig. 6. Two gerbils were exposed to high-intensity (106 dB SPL) tones, and the health of their cochleae was assessed using DPOAEs (Fig. 6a, b). These were substantially reduced over a frequency range that depended on the frequency(-ies) of the damaging tone(-s). This agrees with previous studies on the effect of overstimulation on DPOAEs (e.g., [15]). We note that the damage had the largest effect on the low-SPL responses (Fig. 6g, h), but did not render the cochlea completely passive: intracochlear responses on both the BM (Fig. 6c, d) and in the OHC region (Fig. 6e, f) continued to increase compressively, especially at mid to high stimulus intensities.

**Fig. 7:**
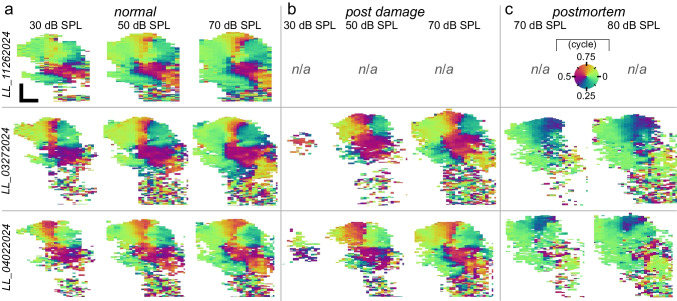
Phase vibrometry difference maps obtained under varying physiological conditions. Series of phase vibrometry difference maps (see also Fig. 5e), calculated as the difference between the mean phase vibrometry maps (averaged for 0.7 ≤ *f*_stim_ ≤ 3 kHz) obtained at a negative (< − 7°) and a positive (> 7°) viewing angle (neg–pos). Data from **a** the healthy, normal cochlea were obtained in three animals (rows). In one animal (top row), each map combines recordings from seven tonotopic locations. For the two other animals, two tonotopic locations were used. Recording from fewer tonotopic locations allowed time to record additional data **b** after exposure to intense sound that damaged (part of) the cochlea, and **c** postmortem. Stimulus levels are per frequency component in the multitone stimulus. Scale bars in (**a**), 50 µm, apply to all maps. Pre- and post-damage DPOAEs and intracochlear (BM and OHC) gain curves for the middle-row (animal ID: LL_03272024) and bottom-row (animal ID: LL_04022204) animal are shown in Fig. 6

The effect of stimulus intensity and overstimulation on the phase flip are shown in Fig. 7. With the reduced number of (tonotopic) recording locations (compared to Fig. 5), the phase difference maps from healthy ears continued to show the 180° phase flip in the core of the ooC, irrespective of stimulus intensity (Fig. 7a). Now, the phase-flip region sometimes extended into the LC, but this seems to be an artifact that can arise when subtracting slightly misaligned maps (see also Online Resource Fig. 2). The sustained cochlear damage did not change qualitatively change the phase difference maps: the phase flip in the OHC/Deiters’ cell region persisted (Fig. 7b). Even at 30 dB SPL, when most vibratory responses had disappeared into the noise floor, signs of the phase flip remained. It only disappeared after the animal had died (Fig. 7c), and most of the intra-ooC motions were in phase with the BM.

## Discussion

We examined in full spatial detail the occurrence of longitudinal motion in the vibratory responses of the ooC. We showed that longitudinal motion was restricted to the OHC, Deiters’ cells and the tunnel of Corti. It was frequency and level-independent but did depend on the ear’s metabolic state; after death, they disappeared. They were, however, robust against the damage induced by acoustic overstimulation. Data were collected in animals of either sex, we have no indications that the observations are sex-dependent.

Pivotal in our analysis is that interferometric methods (such as laser Doppler vibrometry or phase-sensitive OCT) only measure the projection of motion onto their measurement beam. With this, the phase of measured motion would flip (changes 180°) when the angle between this motion and the measurement beam changes from acute to obtuse (or vice versa). In effect, vibrations that move towards (away from) the measurement system under acute measurement angles, move away from (towards) the system for obtuse angles. By placing the cochlea under our OCT system such that the direction of longitudinal motion would be near-perpendicular to the (vertical) measurement beam, it is possible to deduce its presence by looking for this 180° phase flip when systematically changing the angle between this motion and the OCT beam.

Rather than rotating the OCT system relative to the cochlea, we used the natural curvature of the cochlea to vary this measurement angle by comparing responses from adjacent tonotopic locations. This introduces several potential errors in the interpretation of the results. First, variation in measurement angle now co-varies with absolute cochlear location, and either can potentially cause phase effects. We have previously shown that the latter is not the cause for the 180° change in phase [8]. Second, and closely related, is the co-variation of measurement angle and tonotopic location. Due to the traveling wave, the response to a tone will have a different phase at two different tonotopic locations, and their difference may be (by chance) close to 180°. We minimized this potential confound by normalizing all phase responses to the location- and frequency specific phase response on the BM, effectively removing traveling wave effects. Moreover, the wavelengths in this region of the cochlea are 1–3 mm [8], which is substantially larger than the longitudinal distance we used to assess the phase flip. So, even if not compensated for, the traveling wave characteristics would cause a phase difference < 180°. The BM is a suitable choice for such a normalization because its vibrations are expected to be dominated by a simple transverse motion [7]. We chose to use BM phase responses that were averaged over a small region, rather than using the phase response of a single pixel located on the BM. Averaging responses has the obvious benefits (e.g., reduce effect of outliers, less selection bias) but may be inaccurate here because of a phenomenon called phase-leakage [16], which can cause large variation in phase. However, we found that for the BM the within-ROI standard deviation of phase was only 0.07 cycle. Moreover, the fact that we found nontrivial and systematic phase difference across positions and along depths in a single beam argues against any significant effects of leakage, as does the systematic reduction and disappearance of the effects with trauma and death, respectively. Nonetheless, we re-analyzed all responses using the phase of single BM-pixels as a reference, were these pixels where selected based on their strong reflectivity (both absolute and relative to their neighbors). This strategy minimizes potential phase-leakage effects. The results of this re-analysis are shown in Online Resource Fig. 3; its similarity to Fig. 5 verifies that phase-leakage indeed did not affect the analysis of our data.

A final potential error comes from the curvature of the cochlea which causes some “skew” [7, 9], in which a single B-scan is not a single “tonotopic plane” of the cochlea. This is akin to the previous potential confound and can be dismissed for the same reason. Given the small angles re. the longitudinal BM orientation (<|15°|), the height of the ooC (< 150 µm from the BM) and the traveling-wave wavelengths, this skew would introduce, at most, an (OHC–BM) phase difference of a few centi-cycles, much less when viewing angle approaches 0°. So, even when only considering the extreme viewing angles, this is clearly not nearly enough to create a 0.5-cycle phase difference when comparing two “oppositely skewed” tonotopic locations.

It is important to distinguish longitudinal motion from longitudinal coupling [7]. Many cochlear modeling studies have postulated an imported role for longitudinal coupling in the ooC (e.g., [17]). Longitudinal motion and longitudinal coupling are different concepts and, in the context of traveling waves, largely independent. For instance, sea waves show significant longitudinal motion in the absence of longitudinal coupling, whereas torsional waves need longitudinal coupling but show no longitudinal motion. Since the current study reports longitudinal motion and contains no tests of longitudinal coupling, we will limit our discussion accordingly. Longitudinal motion has been predicted by several cochlear models (e.g., [18–20]) and have been measured in gerbils: in vitro in the tunnel of Corti by tracking motions of the tunnel-crossing nerve fibers [21] and in vivo in the OHC region by combining OCT recordings obtained at two different angles [7, 9]. In agreement with these findings, we systematically observed that the 180° phase flip (indicating longitudinal motion) was restricted to the ooC region occupied by the OHC and Deiters’ cells (i.e., its “core”; Figs., 5, and 7). A rather sharp border excludes the BM from this core region. Sometimes, the region extends into the LC, but we interpret that as an artifact of the alignment/calculation method. That is, within the LC, response phase changes rather suddenly with radial location (e.g., Fig. 4), and small misalignments of the phase patterns from different locations causes “edge-effects” in their difference (i.e., the difference between two identical, but misaligned, patterns increases with the pattern’s gradient, which is largest near edges). This effect is most pronounced when comparing only two measurement angles, and its magnitude depends on the values of compared angles (see Online Resource Fig. 2). Averaging across multiple measurement angles (as was done in Figs. 5 and 7, top row) quickly blurs this edge-effect and reduces the apparent phase-flip area within the LC.

It has been suggested that longitudinal motion within the ooC comes from the hydrodynamics of the cochlea, which causes elliptical particle motion in the direction of the traveling wave at some distance away from the BM [18]. Here, the displacements in the longitudinal direction lag the transverse vibrations by 0.25 cycle, which is observed in experiment [7, 8], our data). Indeed, such elliptical motion is found in all fluid waves; it is an inevitable consequence of incompressibility of the fluid. Purely transverse fluid waves do not exist. In sea waves, for instance, the periodic vertical motion of the water, which causes the visible crests and troughs on the surface, is always accompanied by a periodic horizontal motion of the water along the propagation direction (i.e., longitudinal). Thus, all fluid waves show longitudinal motion, and the ratio of longitudinal to transverse motion in fluid waves is determined by the effective depth of the fluid relative to its wavelength λ, or more accurately, λ/2π [18]. Waves on shallow water (“long waves”) are dominated by longitudinal motion; with increasing depth (or decreasing λ), the relative contribution of transverse motion increases. In the limit of deep water (“short waves”), the magnitudes of transverse and longitudinal motion become equal, and the 0.25-cycle phase difference causes the fluid particles to move in circles. Given these well-known properties of fluid waves, it is entirely expected that cochlear vibration should contain a significant longitudinal component. Its dominance, however, is unexpected from the perspective of classical cochlear models. The key issue is the effective depth of the waves.

In the cochlear partition, the effective depth of the fluid is determined by the vicinity of rigid boundaries. In classical cochlear models (e.g*.*, [18, 22]), these boundaries were taken to be the bony wall of the cochlea. Two aspects of our data speak against this. First, the extreme abruptness of the phase flip reveals a profound dominance of the longitudinal motion. Such dominance can only occur with a very shallow effective depth, i.e., a tiny fraction (≤ 1/20, see [8]) of λ/2π. With a wavelength of ~ 2.5 mm, the distance to the rigid boundaries would be 20 μm or less, whereas the distance of the BM to the bony wall in the phase-flip region is 100 μm or more (Fig. 1). The second observation that speaks against the role of the bony wall as the rigid boundary is the immediate post-mortem disappearance of the phase flip. It is unlikely that the mechanical properties of the bony wall would change within minutes after death. Interestingly, both the close (~ 20 μm) vicinity of the effective rigid boundary and its metabolic dependence are consistent with the assumption that the ooC itself provides the rigid boundaries, and that the degree of rigidity (and with it, the effective “depth”) is under the regulatory control of OHCs. This scenario was previously proposed [7] in connection with both the longitudinal motion and the strong variation of vibration pattern (“mode”) with sound intensity [2, 18].

Alternatively, longitudinal motion could result from OHC-mediated cycle-by-cycle changes in the cross-sectional area of the ooC [19], which is believed to provide a mechanism via which cochlear amplification improves the cochlea’s mechanical response [23, 24]. Necessarily, such area changes displace cortilymph, apparently preferentially in the basal–apical direction [20]. Directionality (i.e., towards the base for area decreases) of this fluid flow may arise from the traveling-wave phase characteristics, creating a peristaltic cortilymph pump. This fluid flow could then provide longitudinal coupling within the cochlea. Such directional, longitudinal coupling also comes from the architecture within the ooC’s core, where the apically slanted phalangeal process of each Deiters’ cell and its basally slanted OHC form an asymmetric Y-shaped element. Our data show that the longitudinal motion is restricted to this region. Apparently only the relatively freestanding bases of the OHC (together with the Deiters’ cells) and the fluids in tunnel of Corti have the degree-of-freedom to move in the longitudinal direction. This suggests a correlation between the cellular architecture and the complex motion within the ooC. A computational model of the mouse cochlea supports the hypothesis that the OHC-mediated sensitivity and frequency selectivity of the mammalian cochlea relies on this Y-shaped geometry [25]. On the other hand, if cycle-by-cycle feedback by OHCs would be the main drive behind the longitudinal motion, it is difficult to understand our observation that the phase flip phenomenon (and thus the longitudinal motion) was unaffected by a degree of overstimulation that significantly reduced cochlear sensitivity. It is conceivable that the overstimulation in this study was did not completely abolish the cycle-by-cycle OHC feedback, and that the remaining OHC was still sufficient to produce a large longitudinal motion. This could be tested in a future study by using pharmacological agents that cause a more drastic elimination of OHC feedback.

In our recordings, we found that longitudinal motions dominate the recorded responses in the live cochlea only; the 0.5-cycle phase flip was not observed postmortem. This seems to favor an active role of OHC as a cause of longitudinal motion, where this activity is present at all stimulus frequencies, levels and even in the (partially) damaged cochlea. Whether this OHC role is via cycle-by-cycle feedback, a (static) creation of “rigid boundaries” within the ooC, or some other, not-yet determined, mechanism remains to be resolved. There can, however, be little doubt about the presence of longitudinal motion within the cochlea. Irrespective if it is an epiphenomenon or arises because of the cochlear mechanics that are involved with sensitive sound transduction (e.g., regulation of mechanical properties, cochlear amplification, longitudinal coupling, stimulus to the inner-hair-cell bundles), its amplitude appears to be larger than that of motion in the transverse/radial plane. This warrants caution when interpreting relative 1D motions that include regions within the ooC that are dominated by the longitudinal response (i.e., exhibit the phase flip). Specifically, measured phase differences between structures may simply reflect a difference in the direction of motion rather than a lead or lag for motions that are parallel to the measurement beam.

## Supplementary Information

Below is the link to the electronic supplementary material.

ESM 1 (DOCX 1.26 MB)

## Data Availability

The data presented in this study are available from the corresponding author upon reasonable request.

## Code Availability

The codes used in this study are available from the corresponding author upon reasonable request.

## Declarations

### Disclaimer

These contents do not represent the views of the US Department of Veterans Affairs or the United States Government.
