# Supplementary material for: Longitudinal Vibrations in the Organ of Corti are Limited to Its Core
Source: J Assoc Res Otolaryngol. 2025 Nov 18;26(6):641–53. doi: 10.1007/s10162-025-01017-9 (PMC12698914; doi:10.1007/s10162-025-01017-9)
Supplement: Supplementary file 1 — (DOCX 1.26 MB) [file 10162_2025_1017_MOESM1_ESM.docx]

**Online Resource for:** Meenderink SWF, van der Heijden M, Dong W. Longitudinal vibrations in the organ of Corti are limited to its core. J Assoc Res Otolaryngol. The Resource includes three Figures.


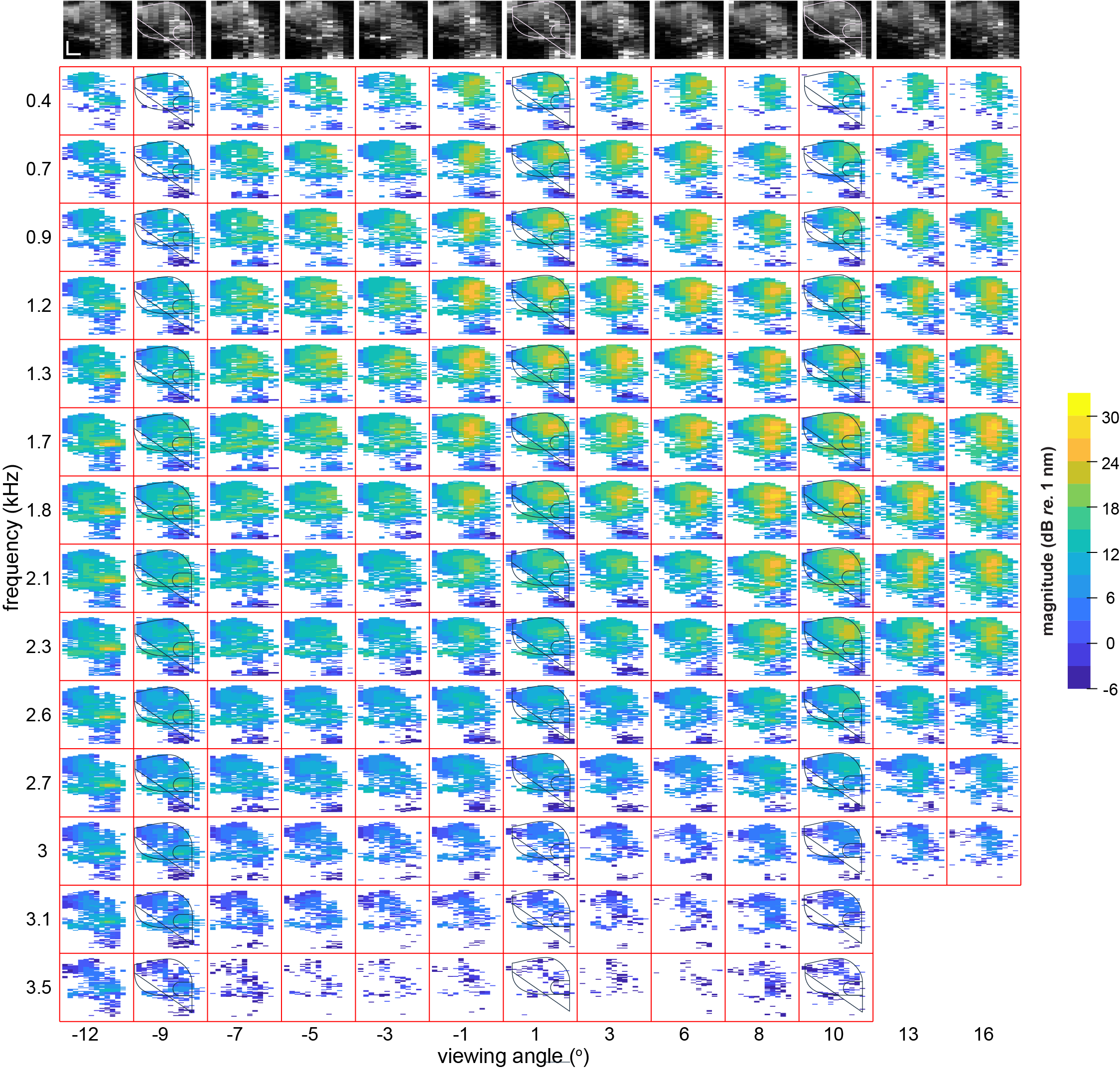


**Online Resource Fig 1:** Collage of radial magnitude vibrometry maps at different frequencies and viewing angles. Data are complementary to the phase maps shown in manuscript’s Figure 4. Each map was obtained for a different stimulus frequency (ordinate) and viewing angle (abscissa). The top row shows the corresponding B-scans for these radial maps. These were created by combining the (depth-resolved) mean intensity of the reflected light during the vibrometry along each A-line that was queried for the map and are identical to those in manuscript’s Figure 4. In three of the columns a coarse structural outline of the organ of Corti is superimposed on the data for visual guidance. Scalebars (50 μm) in upper-left corner apply to all. Stimulus: multitone with 50 dB SPL/frequency component.


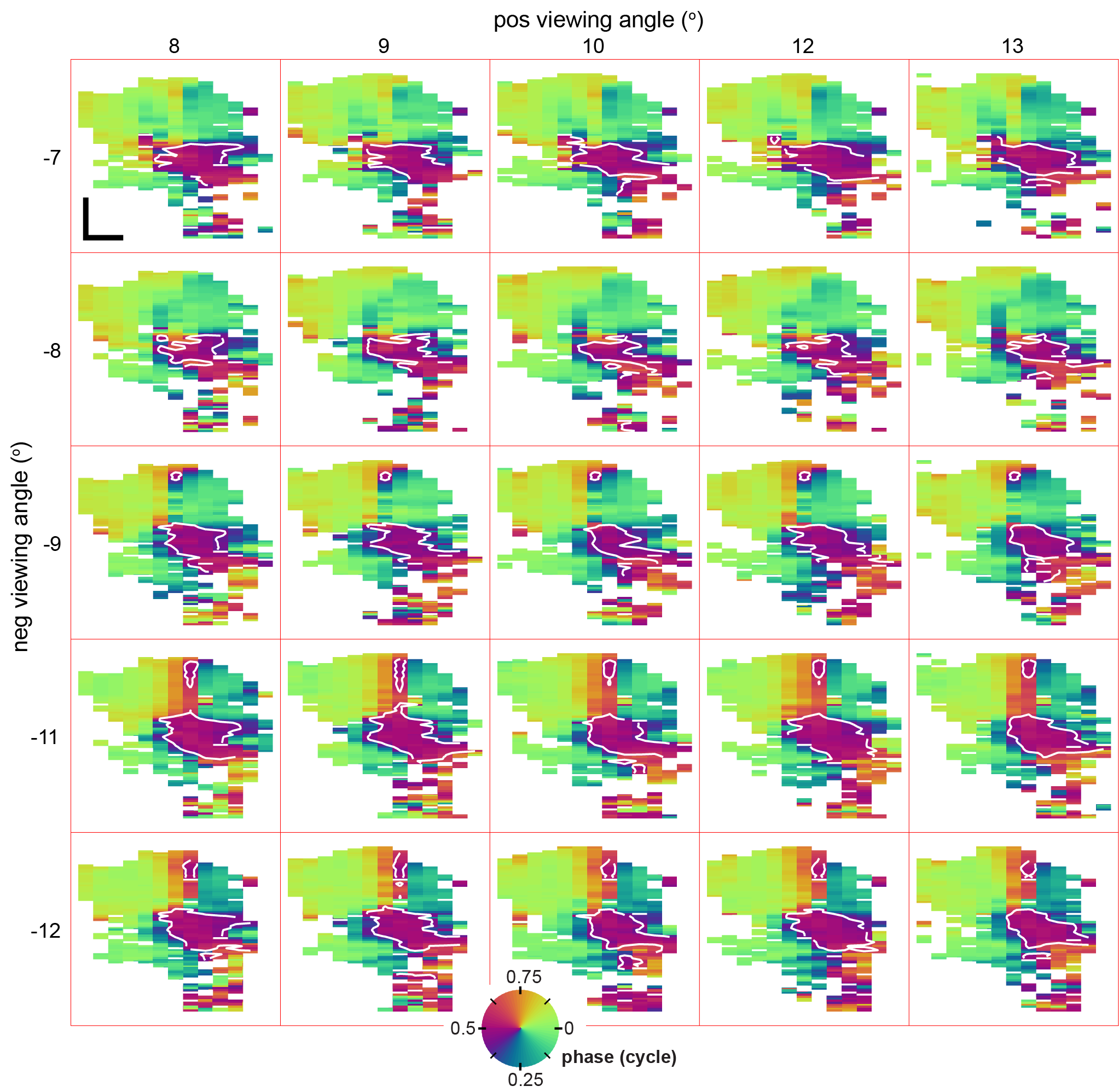


**Online Resource Fig 2:** Grid of vibratory response maps calculated as the phase differences between pairs of two locations with different (positive and negative) viewing angles. Data are from Figure 3 in the manuscript. Depending on the compared angles, the phase difference sometimes shows an "edge-effect" within the lateral compartment, where small misalignment of the two maps, combined with sudden radial phase variation, cause the appearance of a 180^o^ phase flip. Despite this, the similarity between these maps indicate that the exact values of the compared viewing angles are irrelevant. *White lines* are contours at 0.4 cycle. Scale bars: 50 μm.


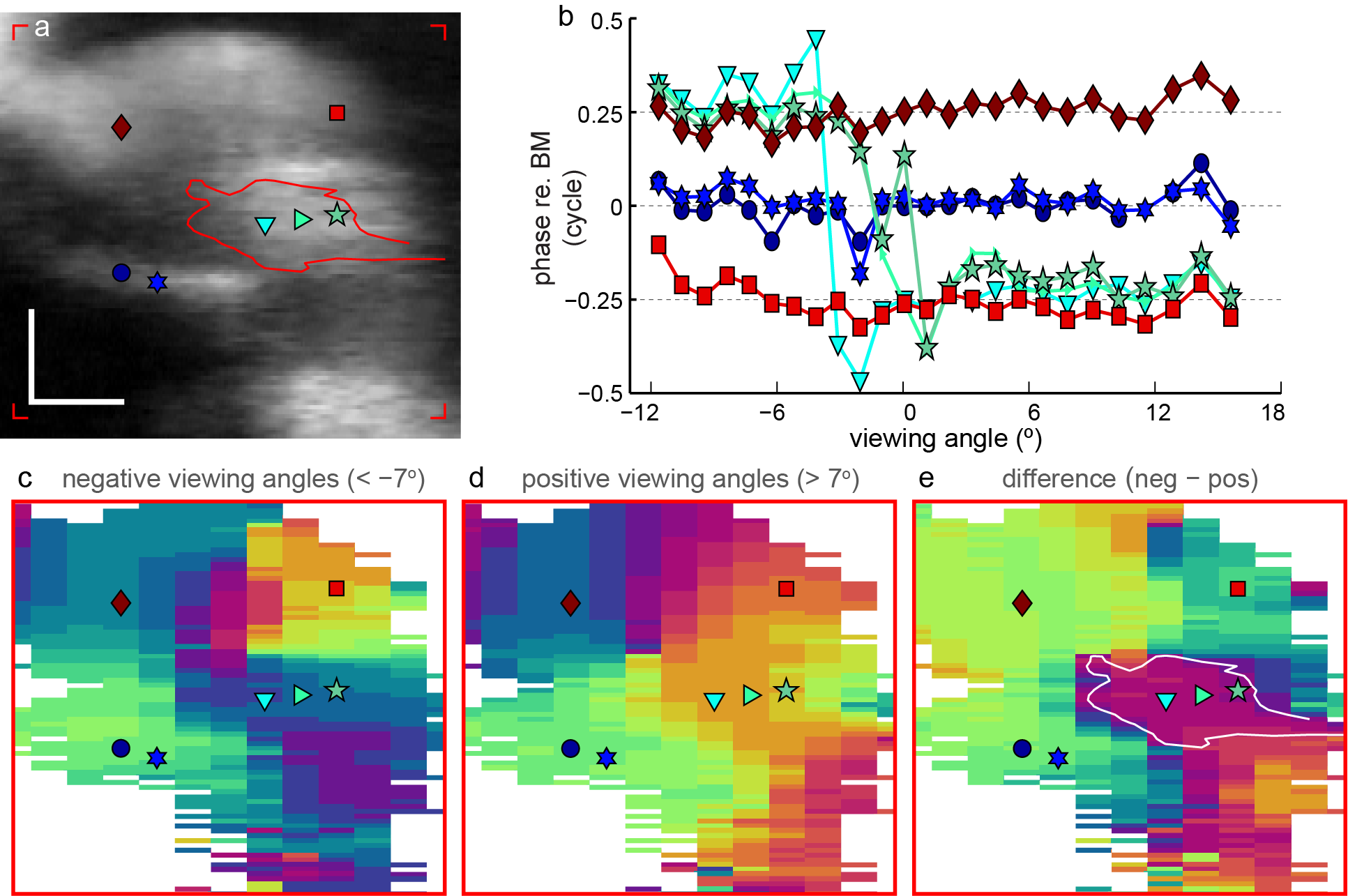


**Online Resource Fig 3:** Localizing longitudinal motion within the organ of Corti. Data and layout are the same as manuscript’s Figure 5. Different from manuscript Figure 5 is how BM phase (which is used as a reference) was calculated. In the manuscript, mean BM phase across a small region-of-interest was used; here the phase of only a single pixel within the ROI was used. The used pixel was the one with largest relative reflectivity (i.e., it was more reflective than adjacent pixels on the same A-line) to minimize potential detrimental effects of phase leakage. **(a)** B-scan, averaged over 25 tonotopic locations after alignment. *Symbols* give pixels for which phase-vs-tonotopic location are shown in (b), *red corner markers* indicate vibrometry region-of-interest. Scalebars: 50 μm **(b)** Phase-vs-viewing angle for marked pixels in (a). Phase is *re.* BM phase and averaged for stimulus frequencies between 0.7 and 3 kHz. *Vertical lines* (marked *N* and *P*) give viewing-angle boundaries used to calculate mean phase vibrometry maps in (d,e). Mean phase vibrometry maps for **(c)** negative (≤-7^o^) and **(d)** positive (≥7^o^) viewing angles, averaged across tonotopic locations and stimulus frequency (0.7 ≤f_stim_≤ 3 kHz). **(e)** Difference between the negative and positive phase vibrometry map. *White line* is a contour at 0.4 cycle, it is shown as a *red line* in (a) where it outlines the Deiters' cells and the basal part of the outer hair cells. *Red boxes* around (c–e) give vibrometry region-of-interest. Stimulus: multitone with 50 dB SPL/frequency component.
